# Predictive role of novel echocardiographic parameter aortic velocity propagation, QRISK3 and Framingham risk score for presence and severity of CAD in Asian patients

**DOI:** 10.34172/jcvtr.2022.25

**Published:** 2022-07-26

**Authors:** Pooja Vyas, Jaykumar Vadodariya, Vijay Kalsariya, Iva Patel, Radhakisan Dake, Kunal Parwani

**Affiliations:** Department of Cardiology, U.N.Mehta Institute of Cardiology and Research Centre (UNMICRC), Civil Hospital Campus, Asarwa, Ahmedabad-380016, Gujarat, India

**Keywords:** Aortic Velocity Propagation, Framingham Risk Score, QRISK3, Coronary Artery Disease

## Abstract

***Introduction:*** Despite having clinical relevance, arterial stiffness is neglected and not routinely used parameter for evaluation of atherosclerosis. This study aimed to investigate the predictive role of simple non-invasive echocardiographic index of aortic stiffness aortic velocity propagation (AVP), Framingham risk score (FHS) and QRISK3 score for presence and severity of CAD.

***Methods: ***This cross-sectional comparative study included 250 patients who required conventional coronary angiogram for stable CAD. The relationship of AVP, FHS and QRISK3 score with CAD were evaluated using spearman’s correlation, logistic regression analysis and ROC curve.

***Results:*** On logistic regression analysis, AVP, FHS and QRISK3 were found significant predictors for the presence and severity of CAD. Inverse correlation between AVP and presence of CAD, number of coronary vessels involved and severity of CAD was observed with *P*=0.001. AVP value≤78 cm/s predicted presence of CAD with 86.4% sensitivity and 84.6% specificity (*P*≤0.0001, AUC=0.948) and≤39 cm/s predicted severe CAD (Syntax score>22) with 66.7% sensitivity and 97.9% specificity (*P*≤0.0001, AUC=0.868). FHS value>10 predicted the presence of CAD with a sensitivity of 33.9% and specificity of 91 % (*P*=0.01, AUC=0.644). QRISK3value>13.4 predicted presence of CAD with 57.1% sensitivity and 87% specificity (*P*≤0.0001, AUC=0.788).

***Conclusion:*** Arterial stiffness parameter AVP is inversely associated with the presence and severity of CAD. AVP and QRISK3 score may be used as a simple bedside tool for risk stratification of patients suspected of having atherosclerotic CAD.

## Introduction

 Atherosclerotic involvement of coronaries, peripheral vascular system and cerebral vasculature is the most common cause for acute coronary syndrome, peripheral vascular disease and cerebrovascular stroke. Atherosclerosis is a generalized pathophysiological process that involves the entire vasculature as well as the coronary arteries. Assessment of the atherosclerotic condition of the large arteries like carotid arteries or the proximal descending thoracic aorta can give an approximate idea about underlying coronary artery atherosclerotic disease.

 Atherosclerosis is a combination of atheromatous and sclerotic changes in the arterial wall. Atherosis means the build-up of fats, cholesterol, and other substances in and on the artery walls, and sclerosis means stiffness of the arterial wall. Imaging modalities like angiography, fluoroscopy, multidetector CT, MRI, and ultrasound can give information about atheromatous changes in arteries. Though larger artery stiffness is recognized as an independent prognostic bio marker which is highly clinical relevant, it is not commonly used in routine clinical practice due to difficulty in evaluation.^1,2^ Arterial stiffness can be measured noninvasively using simple and reproducible echocardiographic parameters like aortic strain (AS), aortic distensibility (AD) and pulse wave propagation velocity.^3^ Increased stiffness due to atherosclerosis causes increased arterial resistance and decreased flow propagation velocity within the arterial lumen.^4^ Several studies have shown the correlation between the extent of coronary artery disease (CAD) and increased aortic stiffness.^5-7^

 Cardiovascular diseases are most common cause of mortality, and even it affects some population a decade earlier, especially during most productive midlife years, depending on lifestyle factors and genetic predisposition. The current traditional risk scores are not sufficient to predict the incidence and severity of coronary artery disease. Coronary angiography is the gold standard test for the evaluation of coronary artery atherosclerotic disease. Other non-invasive predictive tests for coronary artery diseases like nuclear stress sestamibi test, treadmill test, dobutamine stress echo, and PET scan are expensive, time-consuming and have certain limitations. For prediction of presence and severity of CAD, we need simpler and valid parameters, which are easy to perform. Aortic velocity propagation (AVP) can be simple, easily available novel echocardiographic parameter for risk stratification in the evaluation of CAD. The correlation between cardiovascular risk scoring systems (Framingham risk score and QRISK3 score) and the presence or severity of coronary artery diseases (CAD) has not been widely investigated. This study aimed to examine the predictive role of the simple, non-invasive echocardiographic index of aortic stiffness AVP, Framingham risk score and QRISK3 score for presence and severity of CAD.

## Materials and methods

###  Study design and study population

 This cross-sectional comparative study was carried out in the tertiary cardiac care institute between November 2018 to November 2020. Informed consent was taken from all participants. The institutional ethics committee approved the study (UNMICRC/CARDIO/2017/04). 300 patients who needed conventional coronary angiogram were subjected to the following inclusion and exclusion criteria. The study included 250 patients who fulfilled the criteria.

 Inclusion criteria: Patients with suspected coronary artery disease patients with age > 40 years who required conventional coronary angiogram. Patients who gave consent for the study.

 Exclusion criteria: patients with poor echocardiographic image quality, left ventricular ejection fraction ≤ 50%, acute myocardial infarction, prior revascularization (PCI / CABG), patients with moderate or severe valvular lesions, renal failure (serum creatinine > 2 mg/dl), aortic aneurysm, ECG abnormalities like ventricular premature contractions, atrial fibrillation, left bundle branch block and, who refused to give consent for the study.

 All patients underwent routine clinical examination, which included detailed medical history, physical examination, laboratory testing, and assessment of CVD status. Laboratory tests included haemogram, fasting lipid profile, fasting blood sugar, creatinine and hsCRP level. The 10-year risk of MI or death calculation as per Framingham risk score^8^ for hard coronary heart disease, QRISK3 score for having a heart attack or stroke within the next 10 years and QRISK3 (at www.qrisk.org/Open)^9^ healthy heart age was calculated for all patients. All patients underwent echocardiographic examination by two experienced echocardiographers prior to the conventional coronary angiography procedure.

###  Transthoracic echocardiographic examination

 The two-dimensional transthoracic echocardiographic examination was done with the help of S3-1 transducer and echocardiographic machine (iE 33 xMatrix Philips Healthcare, Andover, MA, USA.). Left ventricular (LV) ejection fraction was measured in parasternal long axis view using M –mode and parasternal short axis view at the papillary muscle level, and severity of LV systolic was graded based on American Society of Echocardiography guidelines.^10^

###  Measurement of AVP

**Figure 1:**
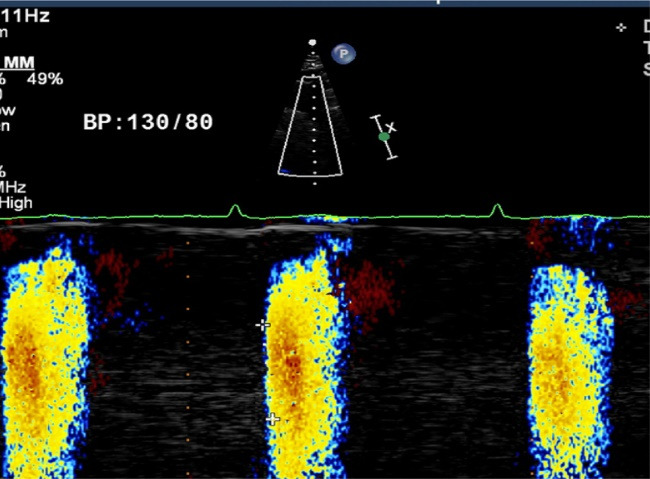
Measurement of AVP.

 The patient is kept in a supine position with neck extension and from suprasternal view, colour M-mode Doppler recordings were recorded from the proximal descending thoracic aorta (DTA) and the cursor was put parallel to the main flow of direction in the proximal DTA. Color Doppler Nyquist limit and m-mode recorder sweep rate was kept between 30–50 cm/s and switched to M-mode with a recorder sweep rate of 200 mm/s; an M-mode spatiotemporal velocity map in the shape of a flame was displayed. The aortic flow propagation velocity was then measured in cm/s by tracing the velocity slope (Figure 1). Beginning and end point of the propagation slope was taken into the calculation. AVP corresponds to the velocity at which the flow was propagating down the artery. The average of 3 measurements was recorded as the AVP value. Intra-observer and inter observer variations were less than 10% which were not significant for APV measurement.

###  Coronary angiography

 Coronary angiography was performed after the detailed echocardiographic examination. Coronary angiography was performed from radial or femoral arterial route using Tiger or standard Judkins catheters respectively. A total of 3-4 views with at least two orthogonal views for the left coronary artery and two views for the right coronary artery were taken. Two independent, experienced cardiologists reviewed the coronary angiography. Coronary luminal stenosis was calculated by visual estimation. Visual estimation of stenosis was performed by comparing percentage of diameter reduction in diseased segment to the disease free proximal reference segment. Left main coronary artery stenosis of 50% or more was considered as significant CAD. Significant CAD was also considered, if there was more than or equal to 50% stenosis in left anterior descending artery (LAD), left circumflex artery (LCX), right coronary artery (RCA) or first order branches of three major coronary arteries. In contrast, insignificant CAD was considered when there was less than 50% stenosis in either one of three major coronary arteries.^11^ These patients were grouped under non CAD group. Syntax score was calculated for all patients.^12^

###  Statistical analysis

 All statistical analysis was done using the SPSS program vs 20. Quantitative variables and qualitative variables were expressed as the mean ± standard deviation and number (%) respectively. We used independent sample *t*-test for comparison of parametric values between two groups. The chi-square test was used to compare categorical variables. The spearman s correlation was used to assess the relation between qualitative variables. Logistic regression was used to predict the presence and severity of coronary artery disease. The predictive diagnostic value of APV, FHS and QRISK3 for coronary artery disease was calculated using the receiver operating characteristic (ROC) curve. Two-tailed *P *value < 0.05was considered as nominally significant.

## Results

###  Clinical, demographic and laboratory parameters

 Clinical and demographic characteristics of patients are presented in Table 1. There were 136 patients in the CAD group and 114 patients in the non CAD group. On matching baseline characteristics, the mean age of patients with CAD was statistically significantly higher than the non CAD group. Waist to hip ratio was found higher in patients with CAD group with a *p*-value of 0.05. Mean AVP, mean 10-year risk of MI or death calculation as per Framingham risk score for coronary heart disease, mean QRISK3 score for having a heart attack or stroke within the next 10 years and mean QRISK3 healthy heart age of both the groups are given in Table 1. Lipid profile andhigh-sensitivity C-reactive protein (hsCRP) values were evenly distributed between both the groups. Cholesterol/ HDL (C/H) ratio was found significantly higher in the CAD group (*P* = 0.03) as compared to non CAD group.

**Table 1:** Baseline characteristics

**Variables **	**Total **	**Non-CAD (N, 114)**	**CAD (N, 136)**	***P*** ** Value**
Age	55.29 ± 11.15	52.14 ± 10.85	57.97 ± 10.86	0.03
Male	144(57.6%)	58 (50.9%)	86 (63.2%)	0.22
Female	106(58.4%)	56 (50.9%)	50 (36.8%)	
**Risk factors**				
Hypertension	128 (51.2%)	50(43.9%)	78 (57.4%)	0.18
Diabetes	66 (26.4%)	24(21.1%)	42 (30.9%)	0.17
Hypothyroidism	10 (4%)	08 (7%)	02 (1.5%)	0.26
Smoking	44 (17.6%)	16 (14%)	28 (20.6%)	0.47
Tobacco	44 (17.6%)	20 (17.5%)	26 (19.1%)	0.99
Family History of coronary artery disease	34 (13.6%)	18 (15.8%)	16 (11.8%)	0.85
**Anthropometric variables**				
BMI(kg/m^2)^	26.88 ± 4.87	25.55 ± 6.80	29.97 ± 8.21	0.07
W-H-Ratio	0.97 ± 0.12	0.93 ± 0.20	0.99 ± 0.14	0.05
**Echocardiography**				
AVP	70.22 ± 33.65	81.37 ± 36.49	60.11 ± 28.18	0.001
**Framingham risk score and QRISK3 score**				
FHS	7.03 ± 8.41	5.22 ± 8.27	8.55 ± 8.36	0.04
QRISK3 score	13.87 ± 11.85	10.10 ± 9.58	17.11 ± 12.81	0.002
QRISK3 Healthy Heart age	60.02 ± 15.19	56.09 ± 14.03	63.81 ± 13.27	0.004
**Lab Parameters**				
Total cholesterol	143.41 ± 40.65	144.29 ± 37.34	142.64 ± 44	0.83
LDL	84.73 ± 33.66	85.44 ± 33.21	84.12 ± 34.89	0.84
HDL	37.18 ± 10.91	39.10 ± 10.52	35.53 ± 11.15	0.08
Triglyceride	121.69 ± 55.84	111.98 ± 54.84	130.08 ± 56.27	0.09
VLDL	25.24 ± 14.68	24.36 ± 11.21	25.99 ± 11.21	0.56
LDL/HDL	2.35 ± 0.93	2.20 ± 0.86	2.48 ± 0.98	0.10
Cholesterol/HDL	4.08 ± 1.11	3.84 ± 1.01	4.29 ± 1.17	0.03
Total lipids	592.55 ± 100.77	580.38 ± 107	603.07 ± 95.88	0.27
HSCRP	1.47 ± 3.01	1.38 ± 3.35	1.54 ± 2.78	0.79

Abbreviations: W-H-Ratio, waist hip ratio; BMI, body mass index; AVP, aortic velocity propagation; FHS, Framingham risk score; LDL, low density lipoprotein; VLVL, very low density lipoprotein; HSCRP, high density C- reactive protein

###  Angiographic Profile of patients with CAD

 Out of 136 CAD patients, 58 patients had single vessel disease (SVD), 42 had double vessel disease (DVD), and 36 had triple vessel disease (TVD). Mean AVP of patients with SVD, DVD and TVD was 72.26 ± 31.83 cm/sec, 56.83 ± 20.34 cm/sec and 41.37 ± 18.25 cm/sec respectively. The inverse relationship between mean AVP and number of vessels involvement was observed, which was statistically significant with a *p*-value of 0.001. For those with significant CAD on CAG, the Syntax score for CAD burden was categorized as low if the score was ≤ 22 and intermediate to high if the score was > 22. The comparison of AVP value between the low Syntax score group (71.14 ± 30.81) and intermediate to high Syntax score group (48.12 ± 26.54) was statistically significant (*P≤* 0.04)

###  Correlation of AVP with risk factors & risk scores

Table 2 represents the spearman’s correlation of AVP with risk factors and risk scores. We found that the inverse correlation exists between AVP and number of vessels blocked (r = -0.365), FHS (r = -0.361), QRISK3 (r = -0.446), Syntax score (r = -0.649) and age (r = -0.395) with a *P* < 0.001 significance.

**Table 2:** Correlation of AVP with major risk factors

**Spearman’s rho**	**Correlation coefficient**	***P*** ** value**
No. of vessels blocked	-0.365	< 0.001
FHS	-0.361	< 0.001
QRISK3	-0.446	< 0.001
Syntax Score	-0.649	< 0.001
Age	-0.395	< 0.001

Abbreviations: AVP, aortic velocity propagation; FHS,Framingham risk score

###  Regression analysis

 Logistic regression analysis represents that AVP (*P* = 0.001), FHS (*P* = 0.05), QRISK3(*P* = 0.004) and Cholesterol/ HDL (*P* = 0.04) ratio were significant predictors of the presence of coronary artery disease. Gender, diabetes and hypertension were found insignificant predictors for severity of CAD. AVP (*P* = 0.03), FHS (*P* = 0.03) and QRISK3 (*P* = 0.03) were the significant predictors of severity of coronary artery disease as given in Table 3.

**Table 3:** Regression analysis for presence and Severity of CAD

**Variables **	**Exp(B)**	**95% CI**	***P*** ** value**
**Presence of CAD**			
Age	1.05	1.02-1.09	0.05
AVP	0.980	0.91-1	0.001
FHS	1.056	1-1.11	0.05
QRISK3	1.06	1.02-1.10	0.004
Cholesterol/HDL	1.47	1.02-2.11	0.04
HSCRP	1.018	0.89-1.16	0.790
**Severity of CAD**			
Age	1.1	1.01-1.18	0.05
Male		0.32-6.30	1.42
Hypertension	3.63	0.69-5.61	0.13
Diabetes	2.73	00.63-11.87	0.17
AVP	0.96	0.92-1.00	0.03
FHS	1.102	1.01-1.20	0.03
QRISK3	1.07	1.01-1.03	0.03
Cholesterol/HDL	1.34	0.67-2.70	0.411
HSCRP	0.927	0.64-1.34	0.685

Abbreviations: AVP, aortic velocity propagation; FHS, Framingham risk score; HDL, High density lipoprotein; HSCRP, high density C- reactive protein

**Figure 2:**
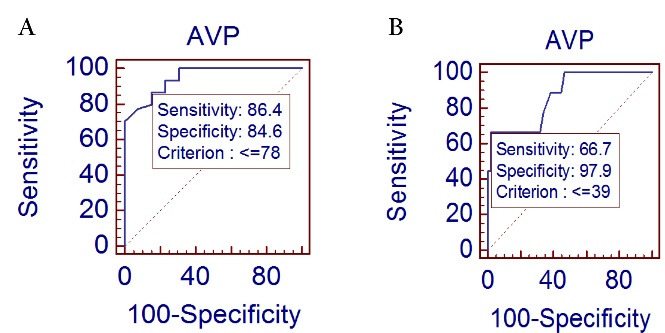
ROC curve of AVP for predicting CAD presence of CAD(A); severity of CAD (B).

**Figure 3:**
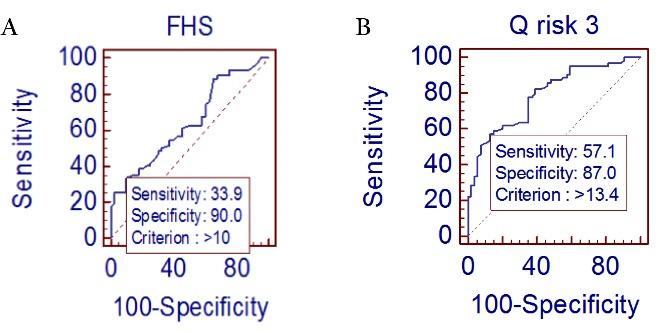
ROC curve of FHS (A) and QRISK3 (B) for predicting presence of CAD.

 ROC curve AVP value ≤ 78 cm/s was used to predict CAD with sensitivity of 86.4% and specificity of 84.6% (*P* ≤0.0001, AUC = 0.948) and ≤ 39 cm/s can be used to predict severe CAD with Syntax score > 22 with a sensitivity of 66.7% and specificity of 97.9%. (*P* ≤ 0.0001, AUC = 0.868) as shown in Figure 1 and 2 respectively. FHS value > 10 shown in Figure 3a, predicted the presence of CAD with sensitivity of 33.9% and specificity of 91 % (*P* = 0.01, AUC = 0.644). Figure 3b represents the QRISK3 value > 13.4 can be used to predict CAD with a sensitivity of 57.1% and specificity of 87% (*P* ≤ 0.0001, AUC = 0.788).

## Discussion

 Overproduction of collagen which is abnormal and reduced quantities of normal elastin causes development of vascular stiffness. This happens due to dysregulation of balance in production and degradation of vascular wall scaffolding proteins, mainly due to stimulation by an inflammatory milieu.^13^ Astudy done by Fazio et al^14^ showed that aortic atherosclerotic plaque detected by TEE is a marker for coronary artery disease with sensitivity and specificity of 90% (negative predictive value 82%, positive predictive value 95%). In addition to conventional cardiovascular risk factors, we need evidence which indicates that information on arterial stiffness helps in prediction and risk stratification of patients for coronary artery diseases. Due to ease and non-invasiveness in AVP assessment, it has gotten attention for assessing aortic stiffness.

###  Coronary artery disease and risk factors 

 In the present study, diabetes and hypertension were found in several cases in the CAD group as compared to the non CAD group, but the difference was not significant. Studies by Sen et al^15^, Vasudeva Chetty et al^7^ and Marakkagari Vamsikrishna et al^16^, also reported the same results on diabetes and hypertension in CAD patients. The BMI was found higher in the CAD group as compared to the non CAD group but the difference was not found statistically significant in our study. Similar findings in relation to BMI were observed in the study by Marakkagari Vamsikrishna et al^16^ In our results, we found significantly higher waist to hip ratio in the CAD group as compared to the non CAD group with a p-value of 0.05.

 In our study, hsCRP was not found statistically different between CAD and non CAD groups. On assessing lipid profiles of CAD and non CAD groups, only total cholesterol to HDL (C/H) ratio was found significantly higher in the CAD group, while other lipid parameters were non-significant in both the groups. In studies reported by Sen et al^15^ Vamsikrishna et al,^16^ there was no statistically significant difference between the lipid profiles of CAD and non CAD groups.

###  Correlation of AVP with CAD

 Our study showed that AVP inversely correlated with presence of CAD which is similar to studies done by Gunes et al,^4^ Vasudeva chetty et al,^7^ Sen et al^15^ and Marakkagari Vamsikrishna et al^16^ Our study reported < 78 cm/s as cut-off value of the AVP for predicting CAD with sensitivity of 86.4% and specificity of 84.6% (*P* ≤ 0.0001, AUC = 0.948). The reported cut-off value of AVP for predicting CAD by studies of Yildrim et al^17^, Vasudeva Chetty et al^7^, Marakkagari Vamsikrishna et al^16^ and Sen et al^15^ was 46.5 cm/s (84% sensitivity and 85% specificity), 47.5 cm/s (76%sensitivity and 72% specificity), 60 cm/s (72.5% sensitivity and 62% specificity) and 60.5 cm/s (90.5%sensitivity and 92.2%specificity) respectively.

 We found an inverse correlation between AVP and number of vessel involvement, which was statistically significant with a p-value of 0.001, which supports the findings by Marakkagari Vamsikrishna et al.^16^

 Our results showed that AVP has a strong and inverse correlation with severity of CAD as measured by calculating a Syntax score (*r* = –0.803, *P* *<*0.0001); significantly lower AVP in those with intermediate to high Syntax score as compared to low Syntax score groups [F (2,66) = 39.30, *P* ≤0.001]. Similar findings were reported by the studies of Marakkagari Vamsikrishna et al^16^ and Vasudeva Chetty et al^7^ while studies done by Sen et al^15^ and Gunes et al^4^ reported no significant correlation between severity of CAD and AVP. These studies used the Gensini score for measuring the severity of CAD, while in our study we used Syntax score which is more commonly used nowadays.

###  Correlation of Framingham risk score and QRISK3 score with the presence and severity of CAD

 There is not much data available on the predictive role of Framingham risk score and QRISK3 score for the presence of CAD as well as severity of CAD using the Syntax score. On logistic regression analysis, FHS and QRISK3 were found significant predictors of presence and severity of coronary artery disease. Our study showed that a cut-off value of 13.4 for QRISK3 score predicted presence of CAD with sensitivity of 57.1 % and specificity of 87%. A study reported by Sayin MR et al^18^ showed that a cut-off value of 7.5 for FHS predicted severe CAD with a sensitivity of 68 % and a specificity of 73% with Gensini score for measuring CAD severity. In comparison to that in the present study, we found the predictive cut-off value of FHS > 10 with a sensitivity of 33.9 % and a specificity of 91 % (*P* = 0.01, AUC = 0.644) for predicting presence of CAD. Our study found that the sensitivity of FHS > 10 for predicting CAD is low 33.9% and AUC is 0.644 which preclude it from considering a good screening tool for predicting the presence of CAD in our patients.

 “Flow propagation velocity” is having relationship with detected maximal velocity points and it is not a true calculation of propagation of fluid between base and apex. So, local pressure gradients in front of the inflow tract can affect results of flow propagation velocity.^19^ Anatomical issues like short-necked, obese individuals, elderly patients and anatomy of aorta can lead to poor echo image quality with suprasternal views and that can cause low reproducibility resulting in intra and inter-observer variability. The results of this study of small sample size need to be confirmed in larger studies.

## Conclusion

 Significant inverse correlation between AVP and presence of CAD, number of coronary vessels involved and severity of CAD was observed in our study. FHS and QRISK3 were found significant predictors of presence and severity of coronary artery disease.

 All findings of our study suggest the role of AVP in the evaluation of arterial stiffness and it can be used in predicting atherosclerotic CAD. Being non-invasive, easily available, time saving and economical, AVP and QRISK3 score may be considered as screening methods for the population at risk and can even be integrated with other CVD risk prediction scores for categorizing patients into low and high risk categories for having atherosclerotic CAD.

## Acknowledgments

 We thank all our patients and clinical staff of our institute for supporting us to complete the work.

## Funding

 This work was supported by U. N. Mehta Institute of Cardiology and Research Centre itself and received no specific grant from any funding agency, commercial or not for profit sectors.

## Ethical approval

 The institutional ethics committee approved the study (UNMICRC/CARDIO/2017/04).

## Competing interest

 All authors have none to declare.
